# *ANGPTL6* Genetic Variants Are an Underlying Cause of Familial Intracranial Aneurysms

**DOI:** 10.1212/WNL.0000000000011125

**Published:** 2021-02-09

**Authors:** Isabel C. Hostettler, Benjamin O'Callaghan, Enrico Bugiardini, Emer O'Connor, Jana Vandrovcova, Indran Davagnanam, Varinder Alg, Stephen Bonner, Daniel Walsh, Diederik Bulters, Neil Kitchen, Martin M. Brown, Joan Grieve, David J. Werring, Henry Houlden

**Affiliations:** From the Stroke Research Centre (I.C.H., V.A., M.M.B., D.J.W.), MRC Centre for Neuromuscular Diseases (B.O., E.B.), and Department of Neuromuscular Disorders (E.B., J.V.), UCL Queen Square Institute of Neurology; Neurogenetics Laboratory (I.C.H., B.O., E.O., H.H.) and Departments of Neuroradiology (I.D.) and Neurosurgery (N.K., J.G.), the National Hospital for Neurology and Neurosurgery, University College London Hospitals NHS Foundation Trust, Queen Square, London; Department of Anaesthesia (S.B.), the James Cook University Hospital, Middlesbrough; Department of Neurosurgery (D.W.), King's College Hospital NHS Foundation Trust, London; and Department of Neurosurgery (D.B.), University Hospital Southampton NHS Foundation Trust.

## Abstract

**Purpose:**

To understand the role of the angiopoietin-like 6 gene (*ANGPTL6*) in intracranial aneurysms (IAs), we investigated its role in a large cohort of familial IAs.

**Methods:**

Individuals with family history of IA were recruited to the Genetic and Observational Subarachnoid Haemorrhage (GOSH) study. The *ANGPTL6* gene was sequenced using Sanger sequencing. Identified genetic variants were compared to a control population.

**Results:**

We found 6 rare *ANGPTL6* genetic variants in 9/275 individuals with a family history of IA (3.3%) (5 missense mutations and 1 nonsense mutation leading to a premature stop codon), none present in controls. One of these had been previously reported: c.392A>T (p.Glu131Val) on exon 2; another was very close: c.332G>A (p.Arg111His). Two further genetic variants lie within the fibrinogen-like domain of the *ANGPTL6* gene, which may influence function or level of the ANGPTL6 protein. The last 2 missense mutations lie within the coiled-coil domain of the ANGPTL6 protein. All genetic variants were well conserved across species.

**Conclusion:**

*ANGPTL6* genetic variants are an important cause of IA. Defective or lack of ANGPTL6 protein is therefore an important factor in blood vessel proliferation leading to IA; dysfunction of this protein is likely to cause abnormal proliferation or weakness of vessel walls. With these data, not only do we emphasize the importance of screening familial IA cases for *ANGPTL6* and other genes involved in IA, but also highlight the ANGPTL6 pathway as a potential therapeutic target.

**Classification of Evidence:**

This is a Class III study showing some specificity of presence of the *ANGPTL6* gene variant as a marker of familial intracranial aneurysms in a small subset of individuals with familial aneurysms.

Three percent of the general population harbor an unruptured intracranial aneurysm (UIA).^1^ Ruptured UIAs cause aneurysmal subarachnoid hemorrhage (aSAH), a devastating condition with high mortality and morbidity and great socioeconomic burden.^2,3^ Despite efforts to discover factors associated with aneurysm formation and rupture, little is known about mechanisms driving aneurysm formation, growth, and rupture as well as the genetic background of this disease. Some inherited disorders, such as autosomal dominant polycystic kidney disease, have been associated with increased aneurysm formation as well as rupture.^4,5^ Increasing evidence exists that genetic factors also play a role in intracranial aneurysm (IA) formation in patients without underlying genetic diseases.^6,7^

Most IAs are acquired lesions arising under hemodynamic stress and associated defective wall response.^8^ Disturbances in angiogenic factors can lead to changes in vessel structure and cerebral artery wall stability. Genes involved in angiogenesis have recurrently been investigated. The angiopoietin-like 6 (*ANGPTL6*) gene is a protein-coding gene belonging to the *ANGPTL* family, which has been associated with regulatory capacities in angiogenesis.^9–11^ ANGPTL6 has been identified as a circulating proangiogenic factor increasing endothelial permeability.^9,12^ A recent publication found significant enrichment in rare coding variants within *ANGPTL6* in patients with familial IA.^13^ They demonstrated a reduction of 50% in the serum concentration of ANGPTL6 in individuals heterozygous for c.1378A>T.^13^

We aimed to externally validate the previous findings, to search for additional rare variants, and to evaluate the rate of rare variants in a large cohort of individuals with family history of IA.

## Methods

### Patient Selection

**Figure 1:**
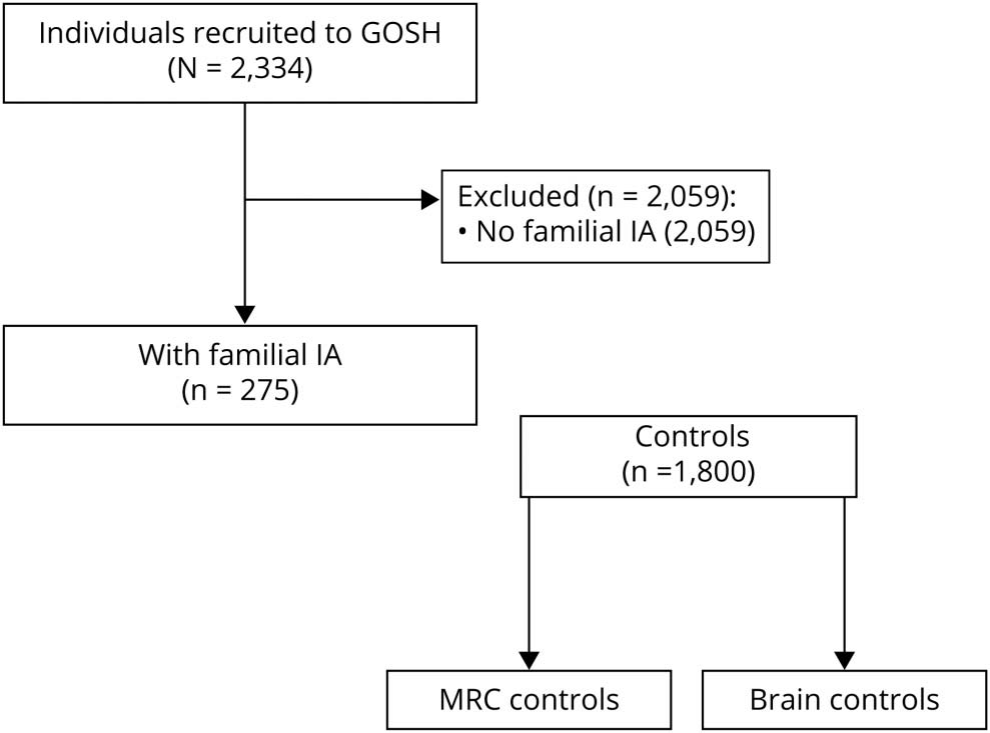
Patients and Controls Selection Flow Diagram GOSH = Genetic and Observational Subarachnoid Haemorrhage; IA = intracranial aneurysm; MRC = Medical Research Council.

For the main analysis, we included patients with available DNA and familial IA where at least 1 affected first- or second-degree relative had IA and was recruited into the Genetic and Observational Subarachnoid Haemorrhage (GOSH) study to evaluate whether previously reported variants would be detected in those with only 1 affected first- or second-degree family member as well (figure 1). Only 1 patient had only 1 affected first- or second-degree relative. Patients with unruptured or ruptured IA could be included in this study. We compare patients with familial IA and the rest of the cohort in table 1 in order to highlight differences. A description of the whole cohort is published elsewhere.^14^ Written informed consent was obtained from all participants, or in case of lack of capacity, from a representative. We did not include patients with perimesencephalic subarachnoid hemorrhage (SAH) (defined by blood distribution mainly or only in the cisterns around the midbrain), absence of intracranial aneurysm, or SAH due to trauma as well as mycotic aneurysms. None of the patients included had been diagnosed with Ehlers-Danlos syndrome, Marfan syndrome, or polycystic kidney disease. IA was confirmed on CT/magnetic resonance angiography or digital subtraction angiography. Because the primary aim of this study was to replicate the association between IA and identifier variants in *ANGPTL6*, and genetic data from affected/unaffected relatives were not collected with the exception of a mother and daughter pair, segregation analysis was not performed.

**Table 1:**
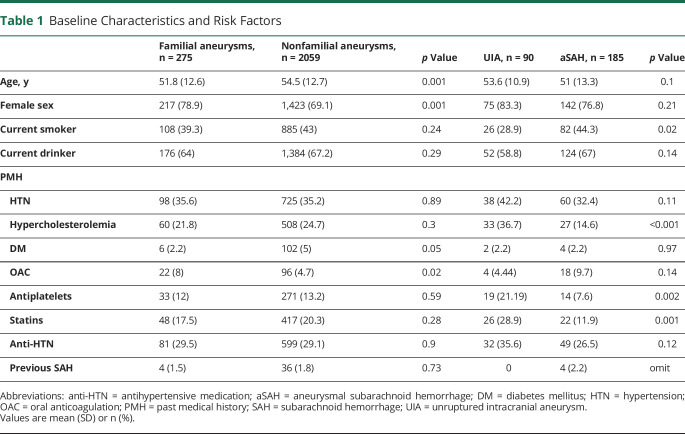
Baseline Characteristics and Risk Factors

The study was approved by the corresponding ethics committee (reference 09/H0716/54).

We used brain biopsy controls from our own laboratory as well as Medical Research Council (MRC) brain exome controls, excluding individuals with known previous stroke (n = 59), to compare the frequency of rare genetic variants found in cases with a control cohort.

The primary research goal is to externally validate and evaluate additional rare variants in the *ANGTL6* gene. This is a Class III evidence study.

### Sanger Sequencing

We extracted genomic DNA and performed PCR to amplify exons 2–6 of the *ANGPTL6* gene followed by Sanger sequencing. Primer sequences can be provided upon request. We cleaned up the PCR product using Exo-Fast, before performing dye-terminator sequencing PCR with BigDye Terminator v3.1 (Thermo Fisher). The sequencing PCR product was cleaned using Sephadex (Sigma Aldrich) before being loaded onto the AB1 3730xl genetic analyzer (Applied Biosystems). We used SeqScape v.3.0 software (Applied Biosystems) for sequence analysis.

In accordance with the previous publication, we filtered out variants with a minor allele frequency (MAF) higher than 1% in the Genome Aggregation Database (gnomAD), v2.1.1.^13^

### Brain Bank Neuropathologically Normal and MRC Controls

Libraries for brain biopsy controls were generated using TruSeq Exome Enrichment Kit (Illumina), according to the manufacturer's protocol, and sequenced on Illumina HiSeq, generating 150bp paired end reads. The average coverage obtained was 36×. Reads were aligned to GRCh37 using Novoalign and variants were called using GATK HaplotypeCaller. Whole exome sequencing data from the MRC brain bank samples were obtained from EGA (EGAS00001001599), mainly sequenced using the Illumina HiSeq 2000 array (ega-archive.org/).

**Figure 2:**
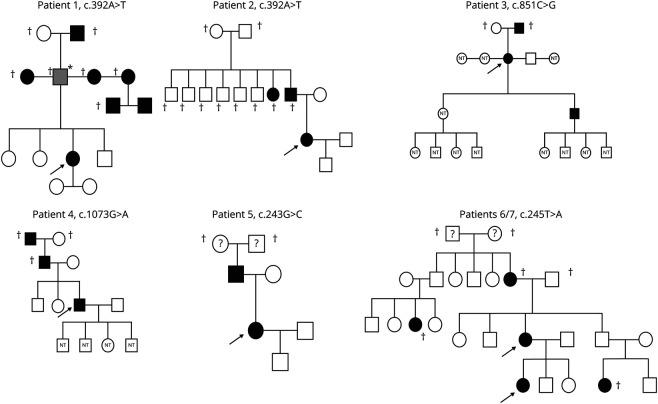
Family Trees of Individuals With Rare Genetic Variants

We compared the MAF of rare variants in our cases with our in-house brain control exomes, the MRC controls, and data available on population databases, specifically gnomAD (gnomad.broadinstitute.org) and Exome Aggregation Consortium (ExAC) (exac.broadinstitute.org) (figure 2).^15^ We then further checked the estimated effect of the according rare mutation using different computation predictive programs. As functional prediction scores, we used PolyPhen-2 and Sorting Intolerant From Tolerant (SIFT). These are used to help interpret the sequence variant.

### Standard Protocol Approvals, Registrations, and Patient Consents

This study received approval from the ethical standards committee on human experimentation for any experiments using human subjects (UKCRN ID-7512, 09/H0716/54).

### Data Availability

Anonymized data will be shared by request from any qualified investigator. EGAS00001001599 can be requested from the European Genome–Phenome Archive.

## Results

We included 275 patients with family history of IA. Of these 275 patients, 185 (67.3%) had had an aSAH. Baseline characteristics and risk factors are summarized in table 1. Comparing familial with sporadic IA, we found that individuals with familial IA were, as expected, younger than their nonfamilial equivalent. Interestingly, individuals with familial IA were more frequently female, and were more frequently on oral anticoagulation, but not on antiplatelets. We did not find a difference in hypertension. In our cohort of patients with family history of IA, 9/275 individuals (3.3%) harbored a rare (<0.01% gnomAD MAF) exonic mutation (figure 2 for the family trees of 7/9 individuals).

**Figure 3:**
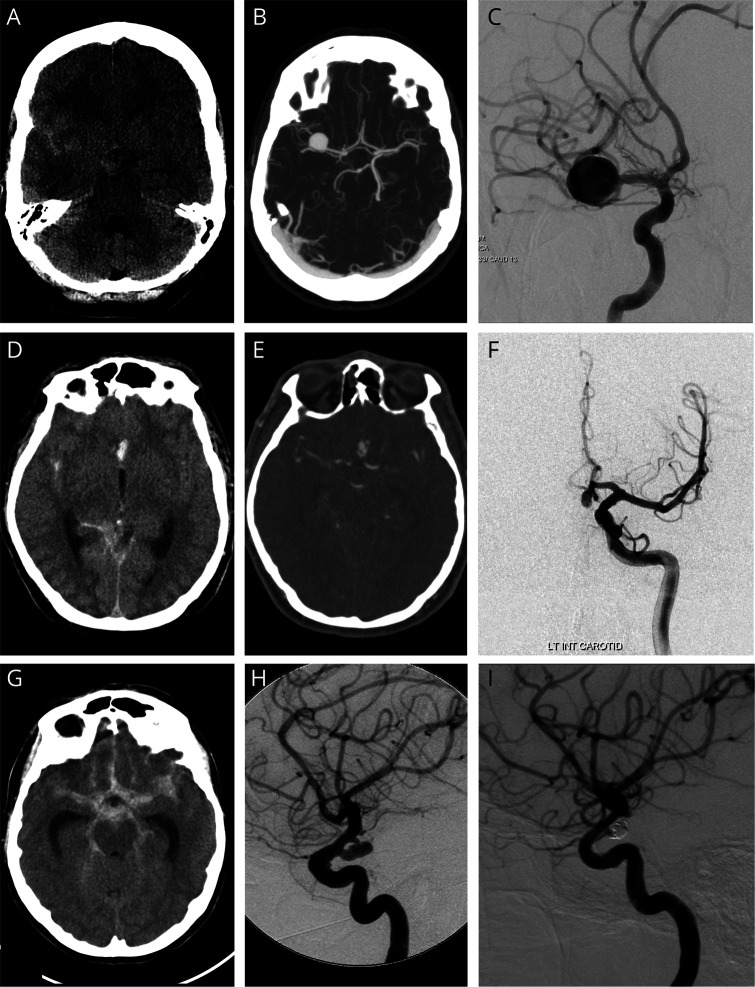
Familial Intracranial Aneurysm (IA) With ANGPTL6 Genetic Variants Had Typical History and Imaging of IA Patient 1: a 48-year-old woman presenting with subarachnoid hemorrhage (SAH) within the right sylvian fissure and suprasellar cistern on the axial noncontrast CT examination (A), from a large right terminal M1 segment right middle cerebral artery aneurysm demonstrated on a maximal intensity projection axial CT angiogram (B), and an oblique view of a selective injection into the right internal carotid artery on digital subtraction cerebral angiography (C). Patient 2: a 41-year-old man presenting with SAH within the right sylvian fissure, anterior interhemispheric fissure, and right quadrigeminal cistern on the axial noncontrast CT examination (D), from an A1–A2 junction aneurysm of the left anterior cerebral artery demonstrated on axial CT angiography (E), and a Towne projection from the selective injection of the left internal carotid artery on digital subtraction cerebral angiography (F). Patient 3: a 57-year-old woman presenting with extensive SAH within the sylvian fissures, anterior interhemispheric fissure, as well as the perimesencephalic and ambient cisterns on the axial noncontrast CT examination (G), and from a posterior communicating segment aneurysm of the left internal carotid artery demonstrated on the lateral projection from the selective injection of the left internal carotid artery on digital subtraction cerebral angiography pre-(H) and post- (I) coil embolization with satisfactory exclusion from the arterial circulation.

We found a total of 13 different nucleotide changes in *ANGPTL6*. Four of these changes resulted in the synonymous amino acid and 3 had a gnomAD MAF above 1% (2 of which were also present in the control group) and were consequently not considered. In the remaining 6 rare genetic variants, we noted 5 different missense mutations and 1 nonsense mutation (table 2). None of these rare genetic variants was present in our control population of 1,800 individuals. Figure 3 demonstrates imaging studies of the aneurysms of 3 of our patients.

**Table 2:**
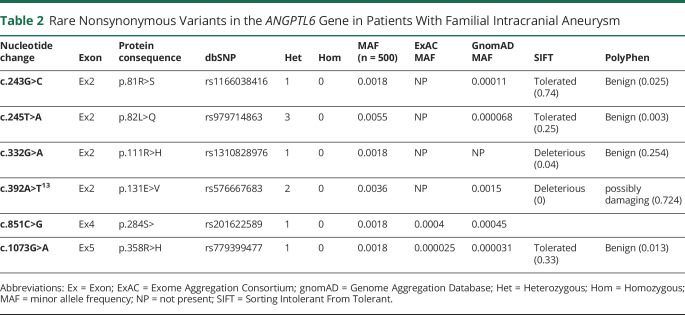
Rare Nonsynonymous Variants in the *ANGPTL6* Gene in Patients With Familial Intracranial Aneurysm

### Previously Reported Variants and Associated Variants

**Figure 4:**
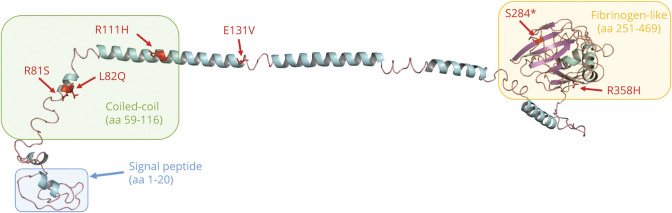
Multiple Alignment Showing Moderate Conservation Across Species and ANGPTL6 Homolog of the Residues Affected by the Variants Identified in our Cohort (Variants Highlighted in Yellow)

**Figure 5:**
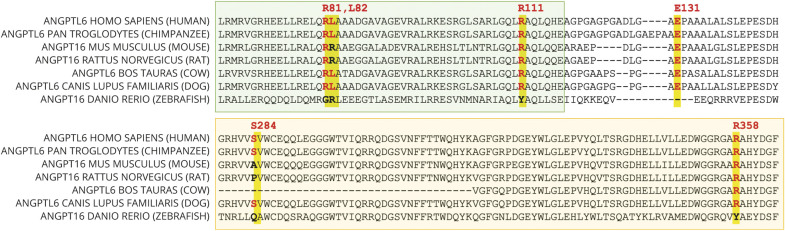
Homology Model of Human ANGPTL6 Protein and Respective Location of the Rare Variants Identified in This Study ANGPLTL6 homology model created using Phyre2^27^ and human fibrinogen beta chain (PDB ID: 3GHG) as the structural template.^28^ Three recognizable domains are present: signal peptide (blue), coiled-coil domain (green), and fibrinogen-like domain (yellow).

We replicated the previous finding of c.392A>T (p.Glu131Val), which has previously been reported as deleterious as per SIFT.^13^ In our cohort, this mutation was present in 2 individuals (MAF 0.004; figure 2, individuals 1 and 2). It was not present in our control population or the ExAC database, but it was present in gnomAD population controls, with a MAF of 0.001. p.Glu131Val is moderately conserved across species, and lies within the linker region between the coiled-coil and fibrinogen-like domains (FLD), but no functional role has been described (figures 4 and 5). We found a previously unreported mutation very close to c.392A>T: c.332G>A (p.Arg111His) in 1 individual (MAF 0.002; figure 2, individual 8). This variant was not present in our control population or ExAC or gnomAD databases and is estimated to be deleterious as per SIFT. The mutation lies within the coiled-coil domain, which is important for the function of angiopoietin-like proteins.^16^ p. Arg111His is moderately conserved across species but no functional role for this specific residue has been described.

### Novel Rare Variants

In addition to c.332G>A (p.Arg111His), which lies in close proximity to the previously reported c.392A>T (p.Glu131Val), we found 4 additional rare genetic variants that have not been reported. Most notably, we found a c.851C>G (p.Ser284*) nonsense mutation leading to a premature stop codon in 1 individual (MAF 0.002; figure 2, individual 3). This variant was not present in our own control cohort; however, it was present in ExAC and gnomAD, with MAF of 0.0004 and 0.0005, respectively. This mutation occurs in Ex4 and is predicted to lead to nonsense-mediated decay (NMD) or production of a truncated protein lacking the majority of the FLD. This stop-gain mutation has been described to result in complete loss of secretion of ANGPTL6 protein in cells.^17^ It is moderately conserved across species.

Another mutation present in our cases lying within the FLD of the *ANGPTL6* gene is c.1073G>A (p.Arg358His), which was again present in 1 individual (MAF 0.002; figure 2, individual 4). This variant was not present in our control group but present in ExAC and gnomAD databases with low MAF of 0.00002 and 0.00003, respectively. p.Arg358His lies within the FLD, which is also important for the activity of angiopoietin-like proteins and is moderately conserved across species, but no functional role of this specific residue has been described.^16^

Finally, we found 2 further missense mutations: c.243G>C (p.Arg81Ser) in 1 individual (MAF 0.002; figure 2, individual 5) and c.245T>A (p.Gln82Leu) in 3 individuals (MAF 0.005; figure 2, individuals 6 and 7; family tree for the third individual not available). Interestingly, 2 of these individuals were related (mother and daughter). These 2 genetic variants affect adjacent residues, and both lie within the coiled-coil domain of *ANGPTL6*. Both amino acids are moderately conserved across species but for neither of them has a functional role been described.

## Discussion

We present a study of the *ANGPTL6* gene in a large cohort with family history of IA replicating 1 of the previously presented variants: c.392.A>T (p.Glu131Val) in the linker region. In addition, we found c.332G>A (p.Arg111His) located very close to this previously described mutation within the coiled-coiled domain. We also found 2 genetic variants in the FLD of the *ANGPTL6* gene, 1 being a nonsense mutation that leads to a stop-gain, which has been described to result in complete loss of ANGPTL6 secretion. None of the genetic variants presented here was found in our 1,800 controls.

Most likely due to the continuous expansion and sometimes merging of databases such as gnomAD and ExAC, some of the previously described variants have now also been described in these control populations. We did not replicate any of the other described variants but found 4 additional ones. All of the rare variants reported here were much rarer in the analyzed control populations, if present at all.

Our study provides additional insight building on the previous study on *ANGPTL6* in individuals with familial IA.^13^ The c.851C>G (p.Ser284*) stop-gain mutation we identified lies in *ANGPTL6* Ex4 and likely results in NMD of the mutant transcript, which could contribute to haploinsufficiency. It is also possible that this mutation leads to the expression of a truncated protein lacking the majority of the FLD, reducing ANGPTL6 protein function or stability. In fact, a previous study reported this variant leads to a complete loss of secretion of the ANGPTL6 protein in cells.^17^ The actual effect of reduced ANGPTL6 secretion remains unknown and warrants further investigation. We also identify novel genetic variants in the FLD and coiled-coil domain of *ANGPTL6*, which might be impairing the proangiogenic function or overall stability of the protein.^16^ Although no function specific to *ANGPTL6* has been established, the FLD is suggested to regulate angiogenic activities.^10,16^ The same is true for the coiled-coil domain. Several functions of this domain have been reported, including activity in polymer formation, molecular recognition, cytoskeletal regulation, and pH sensing.^18^ Based on the information available, a dominant negative mechanism cannot be conclusively ruled out, especially in missense changes. A potential mechanism could be that the mutation alters the protein structural binding, preventing ANGPTL6 signaling. The ANGPTL family also regulates lipid and glucose metabolism and by doing so indirectly influences risk factors for IA formation such as hypertension.^19–22^

As Bourcier et al.^13^ hypothesized, individuals with rare variants in the *ANGPTL6* gene might require additional factors in order to trigger the development of IA. Therefore, heterozygous *ANGPTL6* genetic variants might only exhibit a deleterious effect in combination with certain risk factors such as high blood pressure. Other genes have also been previously reported to play a role in familial IA, such as *ADAMST15* and *PCNT*. *ADAMST15* has been shown to be significantly aggregated in families with IA.^23^ It might be associated with IA formation through abnormal transcription of metalloproteinases.^24^ Two rare variants of the *PCNT* gene have been found in familial IA cases.^25^ Deletions and mutations of this gene cause a type of dwarfism associated with IA in up to 20%.^25^ This indicates the genetic complexity of this disease.

Our study has strengths: we present a large cohort of individuals with family history of IA sequenced using Sanger sequencing. Sanger sequencing remains the gold standard for mutation confirmation. Concordance between Sanger sequencing and whole-exome sequencing has been reported to be 97.3%.^26^ This allowed us to check the variants found in our cases in a control population sequenced by whole-exome sequencing. None of the rare genetic variants found in our cases were present in our own control dataset; if the genetic variants were present in the gnomAD and ExAC control populations, they were significantly rarer, reducing the likelihood of winner's curse.

Our study has limitations: we did not sequence affected or unaffected family members (expect for 2 individuals who were related) as DNA for family members was not available and our aim was to externally validate the previous results of 4 rare coding variants within the *ANGPTL6* gene; information and DNA for relatives were generally not collected as part of the GOSH study. This would be especially of interest as 1 variant was present in both mother and daughter, indicating a potential familial clustering. We included patients with only 1 affected second-degree relative not fulfilling the classical criteria of familial IA. We do not believe that this limits our results as none of the patients harboring a rare variant only had 1 affected second-degree relative. In addition, we were not able to obtain the family trees in 2 individuals; 1 was deceased (c.245T>A; p.Gln82Leu) and the other was not contactable (c.332G>A; p.Arg111His). We therefore cannot make any statements about penetrance in our population. Although we cannot make any statements about penetrance and we refrained from burden analysis due to cases and controls not having been sequenced with the same method, we demonstrated that the rare genetic variants found in our cases are far less frequent or absent in the considered control cohorts. Therefore, a genetic contribution to this disease, including by rare variant in the *ANGPTL6* gene, is highly likely.

We replicated some of the previously reported genetic variants in the *ANGPTL6* gene in a large cohort of individuals with family history of IA. Novel found genetic variants support that *ANGPTL6* loss of function might be a genetic risk factor influencing IA development. These findings remain to be confirmed in an independent cohort. The function of the *ANGPTL6* gene needs to be evaluated. It might offer a target for screening familial IA cases for *ANGPTL6* as well as other genes involved in IA, but also highlights the ANGPTL6 pathway as a potential therapeutic target.

## Glossary

aSAH: aneurysmal subarachnoid hemorrhage
ExAC: Exome Aggregation Consortium
FLD: fibrinogen-like domain
gnomAD: Genome Aggregation Database
GOSH: Genetic and Observational Subarachnoid Haemorrhage
IA: intracranial aneurysm
MAF: minor allele frequency
MRC: Medical Research Council
NMD: nonsense-mediated decay
SAH: subarachnoid haemorrhage
SIFT: Sorting Intolerant From Tolerant
UIA: unruptured intracranial aneurysm
